# Five-year stability of posterior corneal surface after small incision lenticule extraction for high myopia

**DOI:** 10.1186/s12886-022-02463-2

**Published:** 2022-05-28

**Authors:** Yu Zhao, Xue Lin, Zhuoyi Chen, Xingtao Zhou

**Affiliations:** 1 Department of Ophthalmology and Optometry, Eye and ENT Hospital, Fudan University, 83 Fenyang Road, Shanghai, 200031 PR China; 2 NHC Key Laboratory of Myopia, Fudan University, Shanghai, China; 3 Laboratory of Myopia, Chinese Academy of Medical Sciences, Shanghai, China; 4 Shanghai Research Center of Ophthalmology and Optometry, Shanghai, China; 5 Department of Ophthalmology, Dalian Municipal Women and Children’s Medical Center, Dalian, China

**Keywords:** Posterior corneal elevation, High myopia, SMILE, Pentacam

## Abstract

**Background:**

To study the 5-year changes in the posterior corneal surface after small incision lenticule extraction (SMILE) for high myopia.

**Methods:**

Eighty eyes received SMILE was included in this prospective study. They were allocated into two groups based on the spherical equivalent: high myopia (40eyes, -7.49 ± 0.70D) and moderate myopia (40eyes, -4.43 ± 0.87D). Certain points of posterior corneal elevation (the central point (PCE), thinnest point (PTE), maximal point (PME), and in various corneal areas) were evaluated using a Scheimpflug camera (Pentacam; Oculus GmbH, Germany) preoperatively and at 6 months and 5 years after surgery.

**Results:**

All surgeries were completed uneventfully and no ectasia was developed throng the observation. The safety index and efficacy index were 1.14 and 1.03 in the high myopia group, and 1.16 and 1.06 in the moderate myopia group, respectively. Most of the calculated values in the high myopia group showed a slight increase at 6 months but decreased at 5 years. At 5 years postoperatively, the value of the PTE was significantly lower than at baseline in both groups (*P* ≤ 0.047); a statistical difference was also revealed in the PME in the moderate group with slight changes (10.15 ± 3.01 μm vs. 11.60 ± 4.33 μm, *P* = 0.002); no statistical significance was observed in other calculated values (*P* ≥ 0.067). Similarly, no significant linear correlation was noted between changes in all values and the residual bed thickness either (*P* ≥ 0.057).

**Conclusions:**

SMILE causes no protrusion in posterior corneal surface for correction of high myopia at the follow-up visit of 5 years.

## Introduction

With the worldwide application of corneal refractive surgery, cases of iatrogenic keratectasia have been reported. Although the prevalence is very low, it is a serious complication with initial signs of posterior corneal surface protrusion [1, 2]. Therefore, it is of great significance to assess the effect of refractive surgery on the posterior corneal surface. Elevation data, which is independent of orientation and position and free of tear film influence, could represent the posterior corneal surface more accurately than curvature data[3]. Moreover, posterior corneal elevation was considered as the most effective parameter in identifying early ectatic change [3–5].

Recently, the Pentacam (Oculus GmbH, Wetzlar Germany) system has been widely used for assessing corneal topography characteristics. It uses a rotating Scheimpflug slit image camera capturing 25 to 50 images of the whole anterior eye segment from all directions and provides true elevation data. Ha et al. demonstrated that Pentacam is effective in measuring posterior corneal elevation both before and after surgery [6]. Subsequent studies that evaluated the repeatability of the Pentacam also suggested that it provides good precision in assessing the posterior corneal shape [7].

Small incision lenticule extraction (SMILE), a flapless corneal refractive surgery, is considered to be the most minimally invasive surgery. During the procedure, a minimal side cut is made through which the refractive lenticule can be extracted, leaving most of the anterior cornea tissue intact. Investigators compared the total stromal tensile strength in corneas that underwent SMILE, laser in situ keratomileusis (LASIK), and photorefractive keratectomy (PRK) in a mathematical mode, and found that SMILE affects the tensile strength less than LASIK and PRK [8]. However, the impact of SMILE on the posterior corneal surface has not been studied.

In the current study, changes in posterior corneal elevation for high myopia after SMILE were investigated in a 5-year follow-up using the Pentacam, and the results were compared to moderate myopia.

## Patients and methods

### Patients

In accordance with the tenets of the Declaration of Helsinki, the Ethics Committee of Fudan University Eye and ENT Hospital Review Board (Shanghai, China) approved the study protocol (KJ2008-10). Written informed consent was provided by each subject before entering the study.

In this prospective controlled study, 80 eyes of 47 patients undergoing SMILE at the Department of Ophthalmology, Eye and ENT Hospital of Fudan University (Shanghai, People’s Republic of China) were recruited. The patients had no ocular disease other than a refractive error and met the inclusion criteria. Inclusion criteria were as follows: aged more than 18 years when receiving SMILE procedure, stable refraction error in the preceding 2 years, sufficient residual corneal bed thickness more than 250 μm after SMILE, previous sphere equivalent (SE) more than -3.00 D and less than -10.00 D, absent of soft contact lenses at least 2 weeks and hard contact lenses of 1 month and Ortho-K contact lenses of 3 months. Those who met the following criteria were excluded: ocular trauma or diseases, systemic diseases, women in pregnancy or lactation. All patients underwent a comprehensive preoperative ophthalmologic examination, including slit-lamp examination, measurement of uncorrected distance visual acuity (UDVA), corrected distance visual acuity (CDVA), intraocular pressure, and Pentacam HR imaging.

Eyes were categorized into the following groups based on the preoperative manifest spherical equivalent: high myopia group (40 eyes; range, -6.25D to -9.00D; mean, -7.49 ± 0.70D) and moderate myopia group (40 eyes; range, -3.00D to -6.00D; mean, -4.43 ± 0.87D).

### Surgical procedure

The VisuMax femtosecond laser system (Carl Zeiss Meditec AG, Germany) was used to perform all the surgeries. After topical anesthesia, the patient was positioned under the curved contact glass and instructed to focus on the internal target light. The surgeon then achieved correct corneal centration and initiated suction, followed by femtosecond laser scanning. Once the laser scanning was completed, the surgeon inserted a spatula into the cornea, dissected the lenticule interface, and manually extracted the lenticule. The femtosecond laser settings were as follows: repetition rate 500 kHz, 100 µm intended cap thickness, 5.8 to 6.5 mm optical zone (lenticule diameter), 7.3 to 7.5 mm cap diameter, and a 2-mm side cut at the 12 o’clock position. The same experienced surgeon performed all the procedures (XZ).

### Pentacam scheimpflug imaging

All eyes were examined using the Pentacam imaging system. The patient was instructed to position their head on the headrest and fixate on the target light. After attaining alignment, the device captured 25 images and automatically recorded 12,500 elevation points within 2 s. To avoid miscalculations due to poor imaging, the quality of each measurement was shown in the specification window, and only results with “OK” statements were accepted. The examination was repeated if the statement did not meet the requirement (marked yellow or red). Only maps with at least 10 mm of corneal coverage and no deduced data in the central 9-mm zone were accepted.

### Postoperative examination

Follow-up appointments were scheduled at 6 months and 5 years after surgery. Postoperative examinations included Pentacam imaging examinations, slit-lamp examination, measurements of UDVA, CDVA, spherical equivalent refraction and intraocular pressure.

### Data collection

Elevation data of the posterior corneal surfaces were acquired from the Pentacam images. The reference best-fit sphere (BFS) was defined in the center 8.0-mm region of the preoperative data, to ensure it was the same across all images. For points above the reference, values were positive; for points below the reference, values were negative. Calculated values of single points were obtained for the posterior central elevation (PCE), posterior maximum elevation (PME), and posterior elevation at the preoperative thinnest point (PTE) in the central 4-mm area above the BFS. The other 26 determined points in the central 6-mm zone were obtained as follows: 4 points at a 1-mm distance from the center along the 45°, 135°, 225° and 315° meridians (0° was defined as the horizontal semi-meridian on the right, and rotating counterclockwise in both eyes), 8 points at a 2-mm distance from the center at 0°, 45°, 90°, 135°, 180°, 225°, 270°, and 315°, and the other 14 points at a 3-mm distance from the center at 15°, 45°, 75°, 90°, 105°, 135°, 165°, 195°, 225°, 255°, 270°, 285°, 315° and 345°. Posterior corneal elevation in the central 4-mm area and in various optical zones (2-mm diameter, MPE-2 mm; 4-mm diameter, MPE-4 mm; 6-mm diameter, MPE-6 mm) was calculated as the mean value from the points in the corresponding area. Changes in the posterior elevation were found by subtracting the preoperative data from the postoperative data (difference elevation map). The change in elevation was the shift of the posterior corneal surface. Elevation data were recorded in an Excel Spreadsheet (Microsoft Corp, Redmond, WA, USA) for further analysis.

### Statistical analysis

The descriptive results were reported as the mean and the standard deviation. The Kolmogorov–Smirnov normality test and test for homogeneity of variances were performed for all data. The analysis of variance (ANOVA) for repeated measures with the Bonferroni correction was employed to compare pre- and post-operative values. If the data were not normally distributed, the Friedman’s rank test for k correlated samples was used instead of the ANOVA. Bivariate normal analysis was performed before analysis of correlations. Analysis of variance (ANOVA) or the Mann-Whiney U test was applied to compare the differences between groups. The Pearson or Spearman correlation was applied subsequently to determine the association between the changes in posterior corneal elevation and the residual bed thickness (RBT). Statistical analyses were performed using SPSS ver.20.0 (SPSS Inc, Chicago, IL, USA). A *P* value < 0.05 was considered to be a statistically significant difference.

## Results

### Visual outcomes

**Fig. 1:**
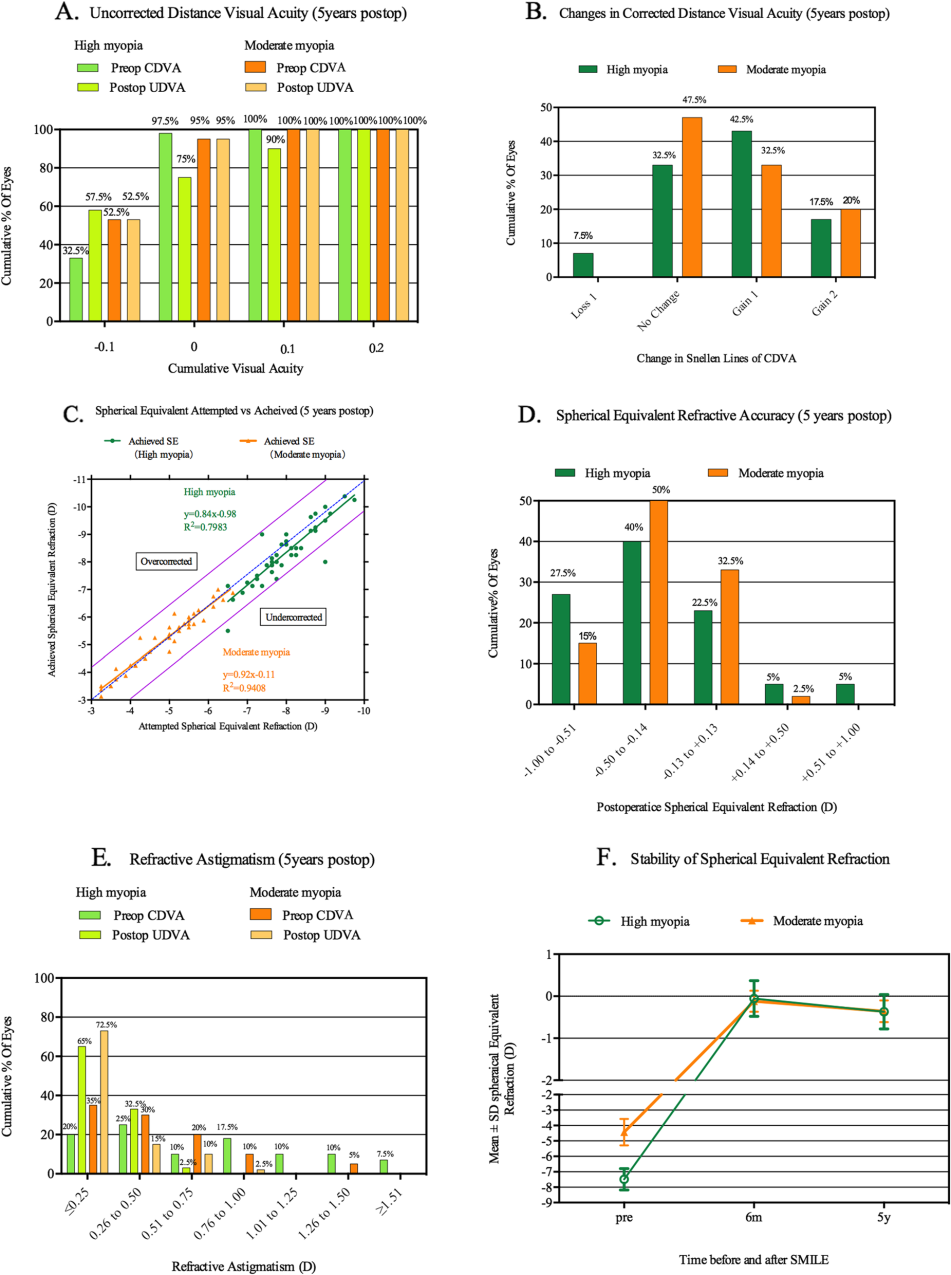
Refractive outcomes at 5 years postoperatively in 80 eyes after SMILE. UDVA = uncorrected distance visual acuity (LogMAR); CDVA = corrected distance visual acuity (LogMAR); D = diopters; Postop = postoperative; Preop = preoperative. **A** Uncorrected distance visual acuity at 5 years postoperatively in both groups. Ninety percent (36/40) of eyes in the high myopia group and 100% (40/40) in the moderate myopia group had a UDVA of 0.1 or better. **B** Changes in CDVA at 5 years postoperatively in all eyes. Only 3 eyes (7.5%) in the high myopia group lost one line of CDVA at the final follow-up compared to preoperatively. The CDVA of other 77 eyes remained stable or improved after SMILE. **C** Spherical equivalent attempted vs achieved (5 years postoperatively). The linear regression formula of high myopia group is “y = 0.84x-0.98 *R*^*2*^ = 0.7983” and of moderate moderate myopia group is “y = 0.92x-0.11 *R*.^*2*^ = 0.9408”. **D** Spherical equivalent refractive accuracy (5 years postoperatively). All eyes were within ± 1.0D; 67.5% (27/40) of eyes in the high myopia group were within ± 0.5D; for moderate myopia, the figure was 85% (34/40). **E** Refractive astigmatism at 5 years postoperatively. 97.5% (39/40) of all eyes in the high myopia group and 87.5% (35/40) in the moderated myopia group were within ± 0.50D. **F** Stability of spherical equivalent refraction. Twenty percent (8/40) of eyes in the high myopia group and 15% (6/4) in the moderate myopia group had diopters changed more than 0.50 D from 6 months to 5 years after surgery

All surgeries were completed successfully, and no complications were occurred either during or after the procedure. Patient detailed information of both groups is shown in Table 1. The safety index and efficacy index were 1.14 and 1.03 in the high myopia group, and 1.16 and 1.06 in the moderate myopia group, respectively. At 5 year postoperatively in 80 eyes, 90% (35/40) of eyes in the high myopia group and 100% (40/40) of eyes in the moderate myopia group had a UDVA of 0.1 or better (LogMAR). Only 3 eyes (7.5%) in the high myopia group lost one line of CDVA at the final follow-up compared to preoperatively. The CDVA of other 77 eyes remained stable or improved after SMILE. All eyes were within ± 1.0D; 67.5% (27/40) of eyes in the high myopia group were within ± 0.5D; for moderate myopia, the figure was 85% (34/40). From 6 months to 5 years after SMILE, 20% (8/40) of eyes in the high myopia group and 15% (6/4) in the moderate myopia group had diopters changed more than 0.50 D. The results are summarized in Fig. 1.

**Table 1:** Patient demographic information of High myopia and Moderate myopia groups (mean ± standard deviation)

	High myopia	Moderate myopia	*P* value
Age			0.550^a^
Mean ± SD	29.03 ± 6.48	29.25 ± 5.74	
Range	20, 40	18, 39	
Preoperative SE (D)			< 0.001^a^
Mean ± SD	-7.49 ± 0.70	-4.43 ± 0.87	
Range	-9.00, -6.25	-6.00, -3.00	
Preoperative TCT (μm)			0.855^a^
Mean ± SD	545.45 ± 34.72	545.35 ± 30.20	
Range	492, 614	498, 606	
AD (μm)			< 0.001^a^
Mean ± SD	139.35 ± 9.90	98.35 ± 15.85	
Range	121, 164	71, 135	
RBT (μm)			< 0.001^a^
Mean ± SD	306.10 ± 33.82	347.00 ± 35.96	
Range	259, 370	283, 406	

*SE* Spherical equivalent, *D* Diopters, *TCT* Thinnest corneal thickness, *AD* Ablation depth, *RBT* Residual bed thickness,.^a^The Mann–Whitney U test

### High myopia

As shown in Table 2, during the first 6 months after surgery, the procedure caused a slight increase in almost all the calculated values of posterior corneal elevation (except the PTE), and statistical differences were noted for MPE-4 mm and MPE-6 mm between the baseline and 6 months postoperatively (*P* ≤ 0.02). On the contrary, the value of PTE after 6 months of SMILE was smaller than at the baseline, and the difference was statistically significant (*P* = 0.023); a similar pattern was also seen at 5 years postoperatively, with *P* = 0.002. No statistical significance was observed in the other calculated values between the baseline and 5 years postoperatively (*P* > 0.067).

**Table 2:** Posterior corneal elevation before and after SMILE in high and moderate myopia group

High myopia	Time point				*P* value		
	Preop	Postop 6 months	Postop 5 years		Preop-6 m	Preop-5 y	6 m-5 y
PCE	1.38 ± 2.60	1.71 ± 3.89	0.55 ± 4.74	0.227^b^			
PTE	3.63 ± 3.55	2.59 ± 4.00	1.40 ± 5.03	0.003^a^	0.023	0.002	1.000
PME	10.78 ± 4.04	12.00 ± 3.93	11.75 ± 3.83	0.102^a^	0.365	0.123	1.000
PCE-4 mm	0.30 ± 0.88	1.08 ± 1.90	0.44 ± 2.64	0.389^b^			
MPE-2 mm	2.05 ± 1.58	2.57 ± 3.09	1.54 ± 3.88	0.317^b^			
MPE-4 mm	-0.64 ± 1.14	0.34 ± 1.79	-0.10 ± 2.50	0.021^b^	0.020	1.000	0.178
MPE-6 mm	-5.12 ± 2.09	-4.53 ± 2.98	-4.35 ± 3.03	< 0.001^a^	< 0.001	0.067	0.091
Moderate myopia	Time point				*P* value		
	Preop	Postop 6 months	Postop 5 years		Preop-6 m	Preop-5 y	6 m-5 y
PCE	1.48 ± 2.35	0.67 ± 4.40	1.00 ± 3.59	0.486^b^			
PTE	4.20 ± 3.41	1.83 ± 5.00	2.38 ± 4.62	0.001^b^	0.002	0.047	0.949
PME	10.15 ± 3.01	10.89 ± 4.14	11.60 ± 4.33	0.001^b^	0.585	0.002	0.088
PCE-4 mm	0.61 ± 0.67	0.69 ± 2.10	1.16 ± 2.10	0.566^b^			
MPE-2 mm	2.04 ± 1.38	1.61 ± 3.22	2.12 ± 3.05	0.578^b^			
MPE-4 mm	-0.11 ± 1.05	0.22 ± 1.91	0.68 ± 1.97	0.083^b^			
MPE-6 mm	-4.31 ± 1.82	-3.47 ± 2.48	-4.05 ± 2.68	0.009^a^	0.006	0.673	0.201

*SMILE* Small incision lenticule extraction, *preop* Preoperative, *postop* Postoperative

*PCE* Posterior central elevation

*PTE* Posterior thinnest elevation

*PME* Posterior maximum elevation

*PCE-4 mm* Mean posterior corneal elevation in the central 4-mm zone of 13 points

*MPE-2 mm* Mean posterior corneal elevation in the 2-mm optical zone as a function of the meridian of 4 points

*MPE-4 mm* Mean posterior corneal elevation in the 4-mm optical zone as a function of the meridian of 8 points

*MPE-6 mm* Mean posterior corneal elevation in the 6-mm optical zone as a function of the meridian of 14 points

^a^The analysis of variance for repeated measures (ANOVA) with the Bonferroni correction..^b^The Friedman’s Rank test for k correlated samples

### Moderate myopia

No statistical significance was observed in PCE between preoperative values and postoperative values (*P* = 0.486). For the PTE, there was a statistically significant decrease both at 6 months and at 5 years after surgery (*P* ≤ 0.047). A significant difference was also revealed in the PME, with a slight increase between preoperatively and 5 years postoperatively (10.15 ± 3.01 μm vs. 11.60 ± 4.33 μm, *P* = 0.002). MPE-6 mm significantly increased at 6 months after SMILE (*P* = 0.006), but no statistical significance was observed between preoperative values and 5-year postoperative values (*P* = 0.673). There was no statistically significant change in the other elevation values during the 5-year follow-up period (*P* > 0.083, Table 3).

**Table 3:** Change of posterior corneal elevation at 5 years after SMILE in high and moderate myopia group

	Change of elevation in high myopia	Correlation with RBT		Comparison between groups (*P*)	Change of elevation in moderate myopia	Correlation with RBT	
		*r*	*P*			*r*	*P*
PCE	-0.83 ± 3.70	-0.211	0.191^b^	0.233^c^	-0.48 ± 2.67	-0.014	0.933^a^
PTE	-2.23 ± 3.74	-0.149	0.359^b^	0.606^d^	-1.83 ± 2.95	0.109	0.505^a^
PME	0.98 ± 2.92	-0.074	0.652^b^	0.508^d^	1.45 ± 3.31	-0.023	0.887^a^
PCE-4 mm	0.14 ± 2.27	-0.183	0.259^b^	0.391^d^	0.56 ± 1.88	0.083	0.611^a^
MPE-2 mm	-0.51 ± 3.11	-0.304	0.057^b^	0.359^d^	0.08 ± 2.47	0.009	0.957^a^
MPE-4 mm	0.54 ± 1.95	-0.156	0.337^b^	0.552^d^	0.79 ± 1.79	0.125	0.442^a^
MPE-6 mm	0.76 ± 1.98	0.290	0.069^b^	0.254^d^	0.26 ± 1.81	0.035	0.832^a^

*SMILE* Small incision lenticule extraction, *preop* Preoperative, *postop* Postoperative

*PCE* Posterior central elevation

*PTE* Posterior thinnest elevation

*PME* Posterior maximum elevation

*PCE-4 mm* Mean posterior corneal elevation in the central 4-mm zone of 13 points

*MPE-2 mm* Mean posterior corneal elevation in the 2-mm optical zone as a function of the meridian of 4 points

*MPE-4 mm* Mean posterior corneal elevation in the 4-mm optical zone as a function of the meridian of 8 points

*MPE-6 mm* Mean posterior corneal elevation in the 6-mm optical zone as a function of the meridian of 14 points

^a^The pearson correlation test. ^b^The spearman correlation test. ^c^The Mann–Whitney U test. ^d^The analysis of variance (ANOVA)

### Correlations

**Fig. 2:**
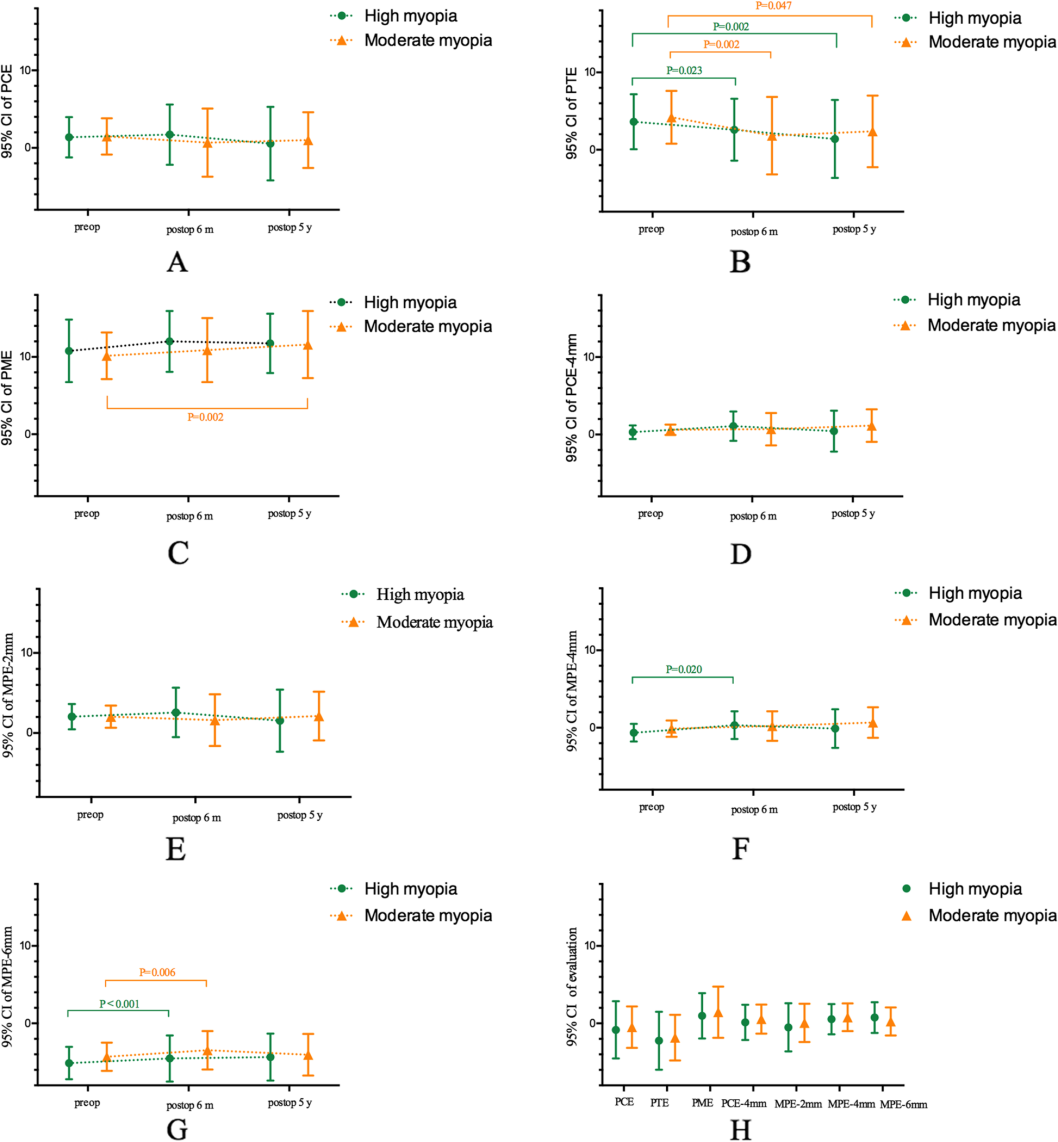
**A-E**: Mean and standard deviation of posterior corneal elevation at different follow-up times in the high and moderate myopia groups after SMILE (80 eyes). Preop = preoperative; Postop = postoperative; PCE = posterior central elevation; PTE = posterior thinnest elevation; PME = posterior maximum elevation; PCE-4 mm = mean posterior corneal elevation in the central 4-mm zone of 13 points; MPE-2 mm = mean posterior corneal elevation in the 2-mm optical zone as a function of the meridian of 4 points; MPE-4 mm = mean posterior corneal elevation in the 4-mm optical zone as a function of the meridian of 8 points; MPE-6 mm = mean posterior corneal elevation in the 6-mm optical zone as a function of the meridian of 14 points. **A** PCE. No statistical significance was observed in PCE between preoperative values and postoperative values (*P* ≥ 0.227). **B** PTE. PTE significantly decreased after SMILE in both groups (*P* = 0.003 for high myopia group; *P* = 0.001 for moderate myopia group). **C** PME. Although no statistical significance was observed in high myopia group (*P* = 0.102), statistical difference was noted in moderate myopia group (*P* = 0.001). **D** PCE-4 mm. No statistical significance was observed in PCE-4 mm between preoperative values and postoperative values (*P* ≥ 0.389). **E** MPE-2 mm. No statistical significance was found in MPE-2 mm between preoperative values and postoperative values (*P* ≥ 0.317). **F**. MPE-4 mm. For high myopia group, there was a statistically significant increase in high myopia group at 6 months after surgery (*P* = 0.020), but no statistically change in 5-year after SMILE (*P* = 1.000). For moderate myopia group, no statistical difference was noted (*P* = 0.083). **G** MPE-6 mm. MPE-6 mm significantly increased at 6 months after SMILE in both groups (*P* < 0.001 for high myopia group; *P* = 0.006 for moderate myopia group). **H** Mean change and standard deviation in the PCE, PTE, PME, PCE-4 mm, MPE-2 mm, MPE-4 mm, and MPE-6 mm at 5 years after surgery in two groups, no statistical difference between two groups was noted (*P* > 0.233)

No significant linear correlations were noted between changes in all values at the final follow-up and the RBT in both groups (*P* ≥ 0.057, Table 3). Data analysis detected no statistical difference in elevation changes between the two groups (*P* ≥ 0.233, Table 3). The results are shown in Fig. 2.

## Discussion

SMILE has advantages over other types of corneal laser surgery as it does not require lifting of the corneal flap, enabling maintenance of corneal integrity to a great extent. In the current study, changes in the posterior corneal elevation were investigated in both high and moderate myopia patients who underwent SMILE in a long-term follow-up (5 years).

Published studies have shown that SMILE can achieve high safety, effectiveness, and predictability for correcting refractive errors [9]. Ağca et al. studied the long-term correction results of SMILE in high myopia, and reported an efficacy index of 0.89 and a safety index of 1.16 [10]. Another study evaluating the 5-year results of SMILE reported that these two indexes were 0.9 and 1.2 [11]. In our study, the index were 1.03 and 1.14, respectively, which were comparable to these studies. In addition, all eyes at the 5-year follow-up were within ± 1.0D, indicating that the correction results were stable with no distinct regression.

The statistical analysis showed that most of the calculated values did not change significantly at 5 years after surgery compared with the baseline, except for the PME. This significant difference was only noted in the PME in the moderate myopia group, and the change was very small and of no clinical significance (1.45 ± 3.31 μm). Byun et al. studied the changes in the PME in eyes that underwent Epi-laser in situ keratomileusis (Epi-LASIK) and observed an elevation change of 2.08 ± 2.29 μm [12]. The authors suggested that this very small difference could not be considered as evidence of corneal protrusion. It is reasonable to conclude, therefore, that there was no forward displacement of the posterior corneal surface in both the high myopia and moderate myopia groups at 5 years after the SMILE procedure. These findings concurred with our previous studies on posterior elevation changes after SMILE in shorter follow-ups [13, 14]. Using the Scheimpflug imaging system, no forward posterior corneal protrusion was observed after SMILE.

It is noteworthy that most of the calculated values in the high myopia group showed a slight increase at 6 months and then decreased at 5 years, but such time-dependent changes were not obvious in the moderate myopia group. Similar changes have also been observed after traditional corneal laser surgeries [15, 16]. The wound healing reaction after surgery with different refractive corrections may partly explain the discrepancy. Previous studies have described that the wound healing process after refractive surgery could last for 6 months, during which epithelial thickening and hypercellular fibrotic stromal scars are produced [17]. In this process, the posterior corneal surface may also be affected and show subtle forward displacement [16, 18]. Besides, with higher refractive corrections, a greater kerotocyte response was pronounced, which leads to a more intense healing response, which may explain why this phenomenon was not observed in the moderate myopia group [19, 20].

Moreover, the changes in the PCE and PTE at the final follow-up after SMILE were both negative in the current study. The findings were similar to those of our previous studies with shorter follow-ups. The average PCE change at 1-year postoperatively in the high myopia and moderate myopia group was -0.27 ± 2.66 μm, and -3.30 ± 3.74 μm, respectively. With a follow-up of 3 years, the elevation changes of these two values were also both negative. This observation was not only noted in our patient database after SMILE, but was also reported by other researchers and after other kinds of corneal refractive surgery. Sy et al. retrospectively investigated PCE changes in patients who underwent LASIK and found a mean postoperative value of 4.55 ± 2.34 μm, which was significantly smaller than the preoperative value of 5.06 ± 2.29 μm [21]. Another study also reported a decrease in PCE from 0.90 ± 2.53 μm to 0.33 ± 4.48 μm after LASIK, and from 1.32 ± 2.58 μm to -1.27 ± 4.62 μm after Epi-LASIK [22]. Contrary to the decreases observed in the PCE and PTE, there was a slight increase in the peripheral area (4-mm and 6-mm zones). Yan et al. investigated the posterior corneal elevation changes after Sub-Bowman Keratomileusis and found that elevation values in the 4-and 6-mm corneal zones were significantly higher than at baseline [15]. A similar progressive forward shift was also noted in the corneal periphery area at 6 months after Epi-LASIK [16]. Researchers have suggested that the hyperopic shift model may account for this phenomenon [15, 16]. As central corneal lamellae are removed after the laser surgery, peripheral lamellae segments relax and become thickened, thus exerting lateral tension at the remaining matrix and producing central flattening and peripheral steepening [23]. In addition, whether keratocyte proliferation, tissue remodeling, and other post-operative tissue responses have an effect on the accuracy of Pentacam measurements is still unclear and should not be ignored.

As shown in the results, no statistical correlation was observed between RBT and elevation changes, which has also been reported in recent publications [15, 16, 24–26]. Previous studies have commented that corneal ectasia is related to the RBT and concluded that posterior elevation changes may increase in eyes with a thinner RBT [27–29]. However, the Orbscan system was used in most of these studies and its reliability in assessing posterior corneal elevation has been questioned; it has been reported that Orbscan overestimated posterior elevation values in eyes that underwent corneal laser surgery [6, 30]. In addition, some of the postoperative eyes had an RBT less than 250 μm [27]. In more recent studies, the standard was strictly followed as all eyes had an RBT greater than 250 μm and Pentacam was used to assess the corneal topography features; thus, elevation changes have been less pronounced and no correlation was observed between the RBT and elevation changes. It should be pointed out that eyes with a preoperative refractive error > -9.00 D were not included in the recent studies, hence future studies could determine whether a correlation exists in eyes with a greater refractive error.

To gain a better understanding of the posterior corneal elevation changes in various regions after SMILE, several calculated values were analyzed. PCE and PTE are values that could reflect the elevation changes of certain points in the central area, as all data were obtained from the same points throughout the study. PCE-4 mm was another calculated value defined as the mean value of 13 determined points, which represented changes in the entire central zone. Additionally, elevation data of optical areas in the central (2 mm), para-central (4 mm), and peripheral (6 mm) zones were also analyzed in this study.

Our study had several limitations: the sample size was relatively small, and both eyes of most patients were included. As ectasia is clearly, in part, genetically determined and typically occurs in both eyes, this was a confounding factor that should be taken into consideration. Another limitation of the current study was the lack of posterior corneal curvature data. Curvature data, which is not constant over the posterior corneal surface, changes from the center to the limbus and through various semi-meridians. Unlike elevation data, curvature data reconstructs the shape of the posterior corneal surface from a different perspective. It is undoubted that these two parameters could represent the posterior corneal surface changes after SMILE more accurately and comprehensively if they had been combined together. Moreover, the preoperative refractive error was limited to less than -9.00 D. Further studies with a larger patient database and in eyes with a refractive error higher than -9.00 D are planned and will provide more information on posterior elevation changes after SMILE.

## Conclusions

In conclusion, SMILE causes no protrusion in posterior corneal surface for correction of high myopia at the follow-up visit of 5 years. More studies in larger number of eyes and longer follow-up are warranted to valid the results.

## Data Availability

The authors confirm that the data supporting the findings of this study are available within the article and its supplementary materials.
